# Platform for combined analysis of functional and biomolecular phenotypes of the same cell

**DOI:** 10.1038/srep44636

**Published:** 2017-03-16

**Authors:** L. Kelbauskas, S. Ashili, J. Zeng, A. Rezaie, K. Lee, D. Derkach, B. Ueberroth, W. Gao, T. Paulson, H. Wang, Y. Tian, D. Smith, B. Reid, Deirdre R. Meldrum

**Affiliations:** 1 Center for Biosignatures Discovery Automation, Biodesign Institute, Arizona State University, Tempe, AZ, USA; 2 Fred Hutchinson Cancer Research Center, Seattle, WA, USA

## Abstract

Functional and molecular cell-to-cell variability is pivotal at the cellular, tissue and whole-organism levels. Yet, the ultimate goal of directly correlating the function of the individual cell with its biomolecular profile remains elusive. We present a platform for integrated analysis of functional and transcriptional phenotypes in the same single cells. We investigated changes in the cellular respiration and gene expression diversity resulting from adaptation to repeated episodes of acute hypoxia in a premalignant progression model. We find differential, progression stage-specific alterations in phenotypic heterogeneity and identify cells with aberrant phenotypes. To our knowledge, this study is the first demonstration of an integrated approach to elucidate how heterogeneity at the transcriptional level manifests in the physiologic profile of individual cells in the context of disease progression.

Rapid advances in the field of analysis technologies for molecular profiling at the single cell level have resulted in unprecedented insights into and discoveries about the cellular machinery and its functional relevance in normal and disease states^1^,^2^,^3^,^4^,^5^,^6^. However, the ultimate goal of relating cellular function with the molecular phenotype and genotype in the same cell remains elusive. Functional heterogeneity exists even in isogenic cell populations and is pivotal in key processes including development, homeostasis, disease etiology, and response to pharmacological agents^7^,^8^,^9^,^10^,^11^. Dissecting the distinct roles intercellular variability plays in disease states, especially as a prerequisite of evolution during the development of cancer^12^,^13^,^14^, holds the promise of novel treatment strategies and efficient drug targets^15^. While several groundbreaking technologies for genotyping, gene transcription, protein expression level, and metabolic profiling at the single cell level exist^16^,^17^,^18^,^19^,^20^,^21^,^22^,^23^, each of them provides only one type of molecular information thus limiting the ability to link differences at the genome or transcriptome level and their phenotypic manifestation in individual cells. Several new techniques for simultaneous characterization of genomic, transcriptomic, and epigenomic molecular profiles of individual cells have recently been reported^1^,^5^,^24^,^25^, yet they represent tools for end-point analysis and do not offer the ability to directly correlate functional parameters of the same cell with its biomolecular profile.

We present an integrated approach for combined profiling of functional and molecular phenotypes of the same individual cells while enabling unbiased, functional readout-based analysis and selection of single cells for downstream analysis. We created a novel integrated platform and approach that combines a technology for respiration rate determination of single cells with a method for harvesting the same cells^26^,^27^, followed by gene expression level analysis on the same individual cells. To demonstrate the biological utility of the approach, we studied how the selective environment of multiple cycles of acute hypoxia affects physiological and transcriptional heterogeneity in pathologic progression represented by premalignant progression of Barrett’s esophagus (BE).

BE is a metaplastic precursor lesion of the esophagus that increases the risk of developing esophageal adenocarcinoma (EAC)^28^. As in many other solid cancers, progressing BE is associated with genomic instability and heterogeneity that evolve in EAC^29^,^30^.

Hypoxia, incurred by the hyperproliferative phenotype of cancer cells that outpaces neovascularization in tumors, is common in many types of solid cancers and is known to play a central role in carcinogenesis, progression, and metastasis^31^,^32^,^33^,^34^,^35^,^36^. In BE, episodes of chronic acid reflux cause the epithelial cells to be exposed to periods of bile, hypoxia, and low pH. It is hypothesized that the interplay between these changes in esophageal environment and immune system response plays a central role in progressing from BE to EAC via selection for the fittest clones that can expand after other competing clones have been eradicated.

We found differential cellular heterogeneity dynamics in the premalignant progression stages in response to acute hypoxia episodes. While we did not detect significant alterations in the cell respiration phenotype among different progression stages, overall gene expression heterogeneity decreased in metaplasia, the early stage of progression, as a result of hypoxia. In contrast, high-grade dysplasia, the late progression stage, showed an increase in gene expression level variability, suggesting an increased ability of the cell population in the late progression stage to adapt and survive under stress. We demonstrate the ability of our approach to identify single cells with aberrant phenotypes by combining physiologic and gene expression profiles.

## Results

**Figure 1:**
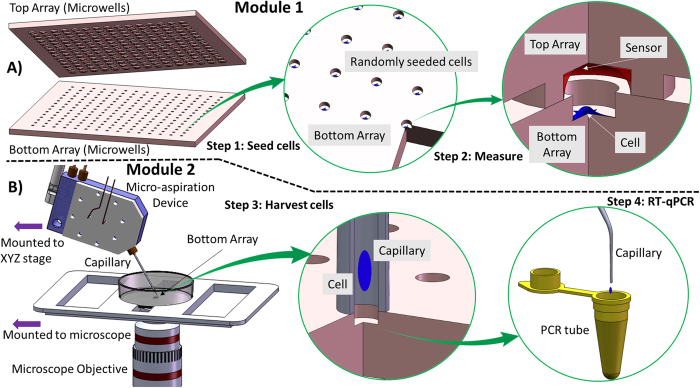
The workflow and device design of the combined functional and biomolecular profiling of the same single cells. The experimental setup consists of two main modules: (**A**) Module 1 for functional profiling of single cells and (**B**) Module 2 for cell harvesting. The functional analysis module consists of a top array and bottom array of microfabricated glass microwells that, when brought together, form an array of hermetically sealed sub-nL microchambers containing one single cell and an embedded luminescent oxygen sensor. The respiration rate of a cell is determined by measuring sensor emission intensity as a function of time. The harvesting Module 2 (**B**) consists of a high-precision membrane-driven micro-aspiration device that enables single cells to be collected and then transferred into PCR-compatible tubes for downstream analysis via RT-qPCR or alternatively, single-cell RNA-Seq of single-cell NextGen sequencing (schematic courtesy of Jeff Houkal).

**Figure 2:**
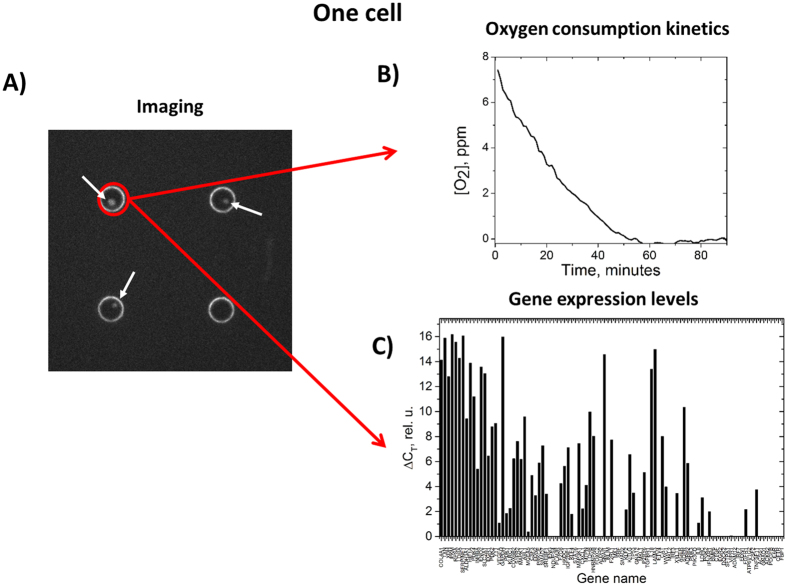
A representative example of the types of information collected in this study from the same single cells using the technology platform. (**A**) Fluorescence image of individual cells with stained nucleus in hermetically sealed wells. Three out of four wells shown contain one metaplastic Barrett’s esophagus cell (arrows, CP-A cell line). (**B**) Oxygen consumption kinetics of the cell in the red circle in (**A**) are shown. The curve represents the absolute concentration of oxygen inside the well as a function of time. (**C**) Expression levels of 96 genes in the same cell as in A and B.

As a demonstration of the utility of our technology (Fig. 1), we performed a study of the integrated dynamics of cellular oxygen consumption and gene transcription heterogeneity in the same single cells in the context of selective pressure conferred by repeated acute hypoxia episodes. The main goal of the study was to gain a detailed insight into the dynamics of cellular physiologic and gene expression heterogeneity under selective pressure. To this end, we measured the rate at which individual cells consume oxygen (respiration rate), then harvested the cells and performed multiplexed gene expression analysis of the same single cells (Fig. 2). We used a panel of four immortalized human esophageal epithelial cell lines representing the metaplastic (CP-A) and dysplastic (CP-B, CP-C, and CP-D) stages in premalignant progression in BE (Supplementary Table 1)^37^,^38^.

**Figure 3:**
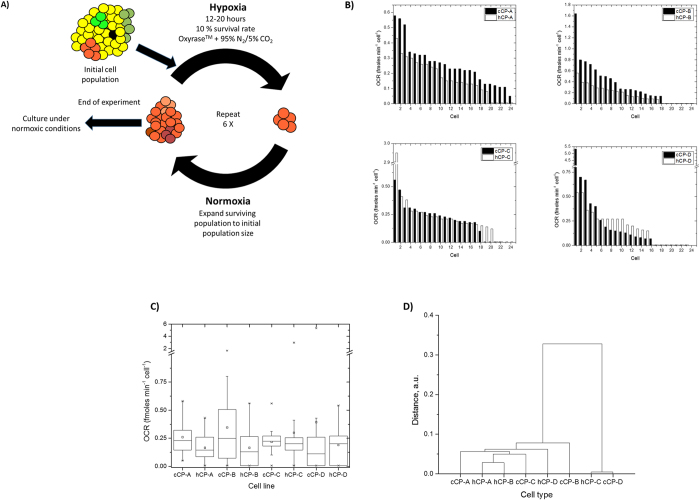
Study of cellular heterogeneity at the functional (respiration rate) and gene expression levels in the same single cells in the context of selective pressure by acute hypoxia. (**A**) Experimental design to generate hypoxia-induced selective pressure on the four cell types (1 metaplastic and 3 high-grade dysplastic with differing genomic lesions). The cells were exposed to six consecutive episodes of hypoxia each time achieving a 10% survival of the initial cell population. Between two consecutive episodes the surviving cell population was expanded to its initial population size under normal culture conditions. (**B**) Single cell oxygen consumption rate (OCR) distributions. The control (“c”) and hypoxia-resistant (“h”) strands of all four cell types showed qualitatively similar distributions. A small fraction of non-respiring cells was present in all cell types except for control CP-A (cCP-A). (**C**) Box plots of the data shown in B. Minor, not statistically significant, differences between the means of the control and resistant strains of CP-A or CP-B cells can be seen. (**D**) Hierarchical clustering based on the OCR data showed similarities between the different strains and cell types, indicating the inability to differentiate cell types only by OCR.

Cells were repeatedly exposed to low oxygen levels (<0.1%) for 6 rounds of 12–22 hours each with the goal of reaching a total surviving fraction of 10% of the starting population size at the end of each hypoxic episode. After episode, the surviving fraction was subsequently expanded to the original cell population size under normal culture conditions before undergoing the next round of hypoxia (Fig. 3A, Materials and Methods). We analyzed 22–24 cells per cell type and cell strain, resulting in a total of 170 individual cells.

### Respiration phenotype characterization

Oxygen consumption rate (OCR) measurements revealed heterogeneous single cell distribution profiles in both strains – control and hypoxia-resistant - of all four studied cell types (Fig. 3B). A comparison of the OCR distribution profiles between the control and hypoxia-resistant strains of all four cell types showed qualitatively similar profiles with a few exceptions. No non-respiring (OCR = 0 fmol/min) cells were detected in the control CP-A strain. Both strains of the three remaining cell types contained a fraction (18–35%) of non-respiring cells. We note that the fraction of the non-respiring cells is markedly higher than the fraction of dead cells (<1%) found in our cell viability tests (Supplementary Fig. S5) and thus cannot be attributed to cell death. This finding suggests differences among the different cell types regarding their response to hypoxia-induced selection. No statistically significant difference was found between the means of the control and hypoxia-resistant strains of all cell types (Fig. 3C, Supplementary Table S2). Hierarchical clustering using the OCR data demonstrated varying similarity levels among the different cell types indicating a limited ability to differentiate cell types and strains based on OCR data alone (Fig. 3D). This finding suggests that changes in cellular heterogeneity as opposed to shifts in the central tendency may be a key measure representative of phenotypic alterations of a population of cells in response to stress.

### Gene expression level analysis

**Figure 4:**
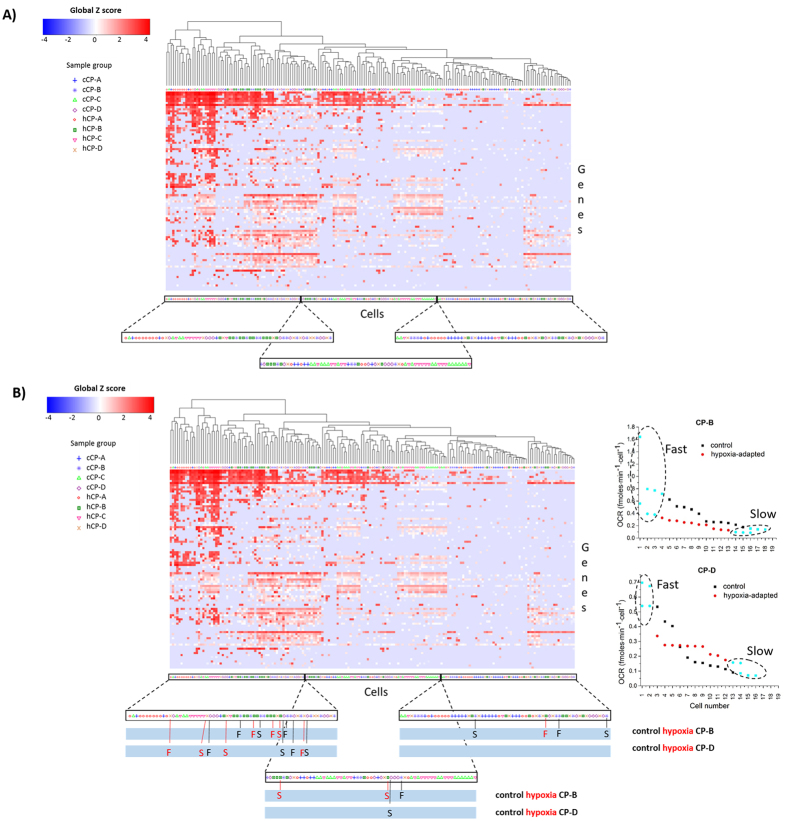
Expression level analysis of a set of 96 genes. (**A**) Heatmap and hierarchical clustering of the gene expression data. The heatmap represents Z scores of expression levels normalized to the global average of the expression levels across all genes. Highly variable expression profiles between and within cell types with no clear clustering by cell type can be seen. (**B**) Combined analysis of OCR and gene expression levels of the same single CP-B and CP-D cells. Cells with fast and slow respiration as indicated in the panels on right are labeled with “F” and S”, respectively on the gene expression heatmap. The control and hypoxia-resistant strands are labeled in black and red colors, respectively. The right panels show the OCR distributions in the corresponding cell types and the dashed regions indicate the fast and slow OCR ranges. The combined analysis shows low correlation between OCR and the gene expression level profiles. For clarity, the lower panels represent zoomed-in areas of the cell type/strain labels.

The genes for analysis were selected to cover several functional categories relevant in the context of hypoxia response, including angiogenesis and glycolysis pathways (Table 1, Supplementary Tables S3 and S4). Hierarchical clustering of the gene expression level data (Fig. 4A) showed an overall highly heterogeneous landscape with only slight clustering by the cell type. Notably, some of the cells of different types or strains exhibited similar target gene expression profiles. Although the number of genes detected in each cell varied markedly, we note that some of the highly expressed genes, such as GAPDH, were detected at similar levels in the majority of cells. This indicates robust performance of our approach towards cell harvesting.

To analyze the variability in gene expression profiles among and within the different types and strands (control vs. hypoxia-adapted) of cells, we first utilized hierarchical clustering (HC) of the gene expression data by grouping the cells into 10 clusters (Supplementary Fig. S6). To quantify the degree of variability within the cell types, we calculated the relative probability of the observed number of cells to fall into a cluster versus what one would expect to observe in the cluster by pure chance, i.e. p_i_ = N_i_/N_total_, where N_i_ and N_total_ are the number of cells in cluster *i* and total number of cells analyzed, correspondingly (Supplementary Materials). The higher the probability ratio the more “enriched” is the distribution, which corresponds to a lower degree of variability. Using this measure we quantified the relative variability (RV) in gene expression profiles in the four cell types by comparing the hypoxia-resistant strains with their corresponding control counterparts (Table 2; Materials and Methods; and Supplementary Table S5). We found that the metaplastic cells (CP-A) showed RV values of 0.59 compared to their normoxic controls. In contrast, the dysplastic cells showed either only a slight decrease in RV (CP-B 0.85, CP-C 0.87) or an increase (CP-D 1.25) (Table 2). These results suggest that the metaplastic and dysplastic cells differ markedly in transcriptional heterogeneity as a result of the exposure to selective pressure via hypoxia.

### Combined OCR and gene expression level analysis

All four cell types displayed heterogeneous both OCR (Supplementary Table S2) and gene expression profiles as assessed by the comparative analysis of the individual cells of each type. Hierarchical clustering of the gene expression data revealed that the cells in the same cluster can show significant differences in OCR. Cells with similar gene expression profiles (in the context of the studied panel of genes) can have markedly differing OCR values and vice versa.

Compared with CP-A and CP-C cells, the CP-B and CP-D cells showed a less clear separation between cells with high and low OCR values in that high and low respiring cells were distributed among several separate gene expression clusters. All CP-B cells with a high OCR belonged to the same expression cluster (marked “F” for “fast” in Fig. 4B), while the slowly respiring control and hypoxia-resistant cells were distributed among different clusters. Moreover, we find that the gene expression profiles of both the control and hypoxia-resistant CP-B strains were more variable as evidenced by the absence of clearly defined clusters in the HC analysis (Fig. 4B). We also observed that the hypoxia-resistant strain of the CP-D cell line showed highly diverse patterns with only weak clustering. The respiration rates were highly variable in even similar gene expression patterns from high to low in both control and hypoxia-resistant strains. The metaplastic (CP-A) cells exhibited the lowest level of variability as determined by analyzing the HC data of gene expression levels and the corresponding OCR values of the individual cells. We observed that fast respirers (high OCR values) and cells with low respiration rates belong to two separate major gene expression clusters (data not shown). We found only 2 cells that were respiring at a low rate and were dissimilar to the two main clusters containing the rest of the cells. The gene expression profiles of the CP-C cell line control and hypoxia-adapted cells grouped into three clusters. Interestingly, the expression profiles appeared to be very similar between the control and hypoxia-resistant strains of the CP-C cells. This finding may indicate that the phenotypic changes that occur as a result of hypoxia are either small or that the changes are reversed under normoxic conditions.

**Figure 5:**
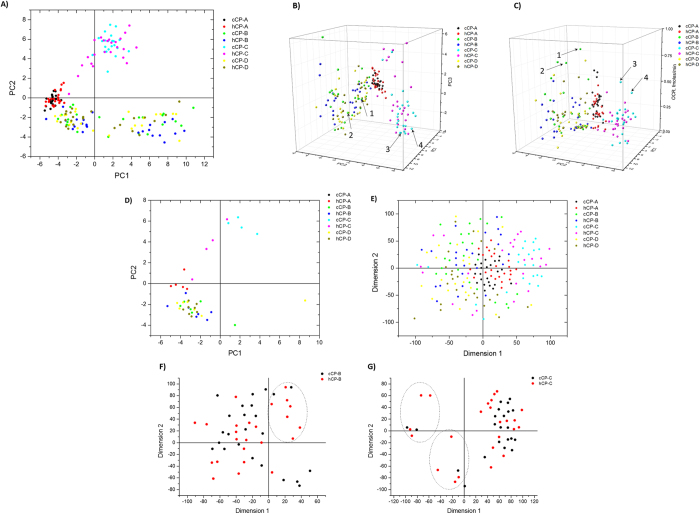
Integrated analysis of respiration rates and gene expression levels. (**A**) Principal component analysis (PCA) of the gene expression data. The loadings of the first two principal components (PC1 and PC2) are shown. The CP-A and CP-C cells form distinct clusters, whereas CP-B and CP-D cells exhibit a similar bi-cluster distribution indicating a more heterogeneous composition of the two cell types. (**B**) PCA of the loading of the first three principal components. (**C**) Combined analysis of the OCR data and PCA identified two control CP-B (green, cCP-B) and two control CP-C (cyan, cCP-C) cells (arrows) that would have been impossible to identify based on PCA only. (**D**) The loadings of the two first principal components of the non-respiring (OCR = 0 fmol/min) cells. (**E,F**) t-SNE analysis of the gene expression data revealed patterns grossly similar to those obtained with PCA, but also identified several new clusters in CP-B and CP-C cells (dashed ovals).

We further conducted principal component analysis (PCA, Fig. 5A) of the gene expression data which led to three important findings. First, the metaplastic (CP-A), the dysplastic CP-C, and dysplastic CP-B and CP-D cells grouped into three separate, clearly defined clusters, respectively. Second, in contrast to CP-A or CP-C cells, both CP-B and CP-D cell types further separated into two sub-populations represented by two distinct clusters. Third, and most importantly, all four cell types exhibited similar gene expression profiles between the hypoxia-adapted strains and their corresponding controls. The hypoxia-adapted cells of all four types appear to fully recapitulate the molecular phenotype of their age-matched control counterparts in terms of the PCA analysis. This finding indicates that even after going through a highly selective process the molecular heterogeneity profile observed in the control cells is recreated and maintained in the hypoxia-resistant strains after the cells return to normoxic culture conditions. It implies that the surviving fraction of the initial population of cells is capable of grossly recapitulating the original gene expression phenotypes of the control cells. Furthermore, the absence of any distinct cell clusters specific only to the hypoxia-resistant strains indicates that no new subpopulations of cells with differing gene expression profiles arise as a result of the selection pressure.

The new ability to simultaneously measure OCR and expression levels of target genes in the same single cells enabled us to analyze how including a functional parameter such as the OCR can be utilized for inquiries into cellular heterogeneity and for identification of cells with aberrant functional and molecular phenotypes. To this end, we replaced the PCA loading data of the third principal component with the OCR data (Fig. 5B, C). We found that several CP-B and CP-C cells with high OCR values were well differentiated from the rest of the cells (arrows in Fig. 5B, C). Furthermore, we identified a cluster of non-respiring (OCR = 0 fmol/min) control and hypoxia-resistant CP-B and CP-D cells that also exhibited highly similar transcriptional profiles (Fig. 5D).

The ability to identify cells with both differing transcriptional and physiological profiles offers a unique tool to find aberrant cells that may indicate what molecular mechanisms underlie differential stress response and survival. We note that when either OCR or gene expression data are considered separately, identification of such cells would not be possible due to the similarity among gene expression or OCR data. This demonstrates the utility of our technology to identify aberrant cells within a population of cells.

Next, we analyzed the gene expression data utilizing the t-distributed Stochastic Neighbor Embedding (t-SNE) method^39^. The t-SNE method is different from PCA in that it captures non-linear relationships between data points and maps them into a 2D or 3D space. The t-SNE method has been demonstrated to reveal the presence of subpopulations of cells in single cell transcriptome^4^ and mass spectrometry^40^ studies. The analysis revealed qualitatively similar results as PCA, but highlighted several new details (Fig. 5E). The hypoxia-resistant and control strains of each of the four cell types clustered similarly. However, in contrast to the PCA analysis, both the CP-B and CP-C cells showed a sub-population of cells that separated from the main cluster (Fig. 5F, G).

## Discussion

Expanding the types of information that can be collected about the same single cell has been a major thrust in the field of single cell analysis. Different types of data about the same cell holds the promise of not only gaining novel insight into the cellular function, but also establishing reliable networks of interactions between the events taking place at the cellular and biomolecular scale. The technology presented for integrated phenotypic characterization at the individual cell level enables characterization of functional and biomolecular phenotypes of the same single cells. It is a versatile tool to explore cells with aberrant combined phenotypes. The method is agnostic to cell types and is versatile for use with a variety of other biological readouts, including intracellular fluorescence markers for DNA, ion concentrations, and cellular organelles. For example, combined extracellular pH/oxygen^41^ or oxygen/glucose^42^,^43^ sensors can be utilized for multi-parameter analysis of metabolic profiles at the single cell level. The method may be combined with advanced single-cell end-point analysis techniques such as next generation sequencing and/or proteomic profiling for insights into the cellular machinery under normal conditions and in a disease state. While high-throughput single-cell NGS approaches exist^1^,^5^,^6^,^9^,^16^, they are based on random selection of individual cells and none of them offers the ability to associate the cellular and the biomolecular phenotype of the same cell. Our method offers a means to overcome this limitation by enabling combined analysis of the cellular and biomolecular phenotypes of the same cell.

Furthermore, while the platform is primarily designed to work with individual cells, it can be adapted to the analysis of more than one cell per well by increasing the number of cells per well^27^. The number of cells can be increased by simply increasing the cell seeding density and/or increasing the well size. In this way, the effects of cell-cell communication on cellular phenotypes may be studied as a function of the number of interacting cells^27^ with the ability to extract individual cells for downstream analyses^44^. Phenotype and genotype analyses of cell populations ranging from a few cells to hundreds and thousands of cells may be performed using the same approach. The system throughput may be improved by increasing the number of wells on the bottom array. However, with the increasing number of wells additional technical requirements for hermetic sealing and force distribution will arise.

To fully automate our platform, we have automated in the past the process of lid removal after drawdowns as well as fully automated the cell harvesting step. Our experiments have shown that even when using a combination of enzymatic digestion of integrins and shear flow to harvest adherent cells with minimal cell stress as it was done in this study, marked cell-to-cell variability in how easily a cell can be retrieved due to varying cell adhesion strength poses a major challenge. During cell harvesting, one typically faces the difficulty of keeping the cell membrane intact while retrieving the cell so as to not lose the cell contents for gene expression analysis, while performing the retrieving step as quickly as possible to avoid potential changes in the gene expression profile. As a result, the cell harvesting step becomes more complex and requires on-the-fly fine tuning of the cell retrieval parameters such as the suction flow rate, distance between the pipette tip and the cell etc. Nevertheless, the two steps of the experimental workflow – oxygen consumption rate measurements and gene expression profiling - could be automated by employing advanced computer vision-based algorithms and additional hardware for cell harvesting. Alternatively, some gene expression or other analyses with low multiplexing could be performed on the chip thus avoiding the cell harvesting step completely.

To demonstrate the capabilities of our technology platform and to highlight the utility of characterizing both the functional and molecular phenotype at the single cell level, we combined measurements of oxygen consumption rate with expression levels of a panel of 96 genes of the same single cells and were able to identify cells with aberrant combined phenotypes. While the functional relevance of these cells in the context of population response warrants further studies, this result demonstrates the ability of the approach to provide insight into cellular heterogeneity at both the functional and biomolecular level. Conceivably, the platform may be used for drug screening assays with the goal of identifying individual cells with aberrant response to treatment and underlying molecular mechanisms. Clearly, it would be desirable to increase the number of analyzed cells per cell type to hundreds or more for increased statistical significance and detection sensitivity for aberrant cells. However, despite the relatively low number (20–24) of individual cells analyzed per cell type, our study has shown that marked differences in response variability to hypoxia among the different stages of premalignant progression exist. It is likely that an increased sample size may reveal other sub-populations of cells, either as new clusters in the PCA analysis or sub-sets of the reported clusters. We note that although the total number of individual cells analyzed appears low, the number of readout parameters (expression levels of 96 genes +OCR) per cell is considerable. For example, it has been demonstrated that cell sub-populations can be robustly identified on the basis of as low as 17 single cells by analyzing expression levels of 48 genes in a total of 159 cells^45^. Therefore, it is conceivable that our approach offering the functional OCR readout in addition to 96 genes is well suited to detect differential phenotypes in cell sub-populations. We recognize, however, that the detection sensitivity and specificity depend strongly on the choice of genes and functional parameters. Validation of these results in a greater number of cells using a high throughput approach of the next generation of this instrument (currently in development by our group) will improve our understanding of this phenotypic variability. Our technology platform would enable a future, in-depth inquiry into single cell heterogeneity in the context of premalignant progression.

The study demonstrates our platform for combined analysis of functional and biomolecular phenotypes of the same single cell and the findings demonstrate that while hypoxia-induced selective pressure reduces phenotypic variability in the early progression stage, it remains the same or increases in the more advanced stage. The results suggest that the hypoxia resistant cells are able to recapitulate the phenotypic variability in both respiration and gene expression levels similar to that observed in control cells.

## Materials and Methods

### Device fabrication and preparation

Lids and microwells were fabricated in fused silica substrates using wet-etch lithography as described elsewhere^46^. After fabrication the wafers were coated with a layer of poly(para-xylylene) (Parylene C), a biocompatible polymer with low gas permeability, that serves as oxygen barrier facilitating sealing. The wafers were then diced into rectangular pieces each corresponding to a 15 × 15 array of wells or lipped lids. Following dicing, the substrates were placed into a custom Teflon holder and positioned into a 250 mL glass beaker containing a 1% micro-90 solution (Sigma-Aldrich, St. Louis, MO) for cleaning. The beaker with the substrates was sonicated for 30 minutes to help loosen any solid particles attached to the surface. After sonication the substrates were cascade rinsed with purified water and then sprayed with 70% ethanol before leaving them to air dry in a laminar flow hood. The cleaning step is important to ensure that there are no particles that may interfere with producing a hermetic seal during drawdown.

Next, the wells in the substrates were deposited with a mixture of extracellular matrix (ECM) proteins containing 19.5 μl of poly-L-lysine, 3.0 μL of laminin, and 7.5 μL of fibronectin from corresponding stock solutions (laminin, catalog # 114956-81-9, poly-l-lysine solution, catalog # P4707, fibronectin from bovine plasma solution, catalog # F1141-1MG, all three from Sigma-Aldrich) utilizing a non-contact piezoelectric liquid dispensing robot (Rainmaker, au301, Aurigin Technology Inc., Phoenix, AZ). The composition of the ECM protein mixture was determined experimentally to be optimal to facilitate cell adhesion to the substrates for all 4 cell types used in the study. After deposition with the ECM mixture the well substrates were stored at 4 °C until further use. We note that while the treatment of the substrates with the ECM proteins mixture was optional, it resulted in better and rapid adhesion of all four cell types to the substrates. After seeding, cells are allowed to adhere to the bottom of the wells for 10–15 minutes followed by washing the chip with a fresh cell growth medium to remove excess cells that are not in the wells. More details on wells and lids arrays preparation can be found in Supplementary Materials. The feasibility to achieve hermetic sealing of the wells was verified by purging the cell media with varying mixtures of oxygen and nitrogen, while monitoring the sensor response over a time period of 50–80 minutes. The presence of hermetic sealing in a well was confirmed if the sensor intensity did not change during the entire test.

### Sensor readout and calibration

A self-referencing, ratiometric chemosensor^41^ was used to quantify oxygen flux of single cells^27^. Sensor emission intensity was monitored for the entire array during each experiment so the intensity change of each distinct well could be evaluated both in real time and during offline data analysis. The sensor emission intensity can be normalized against the rhodamine 123 channel as a self-reference, making the intensity quantification independent of sensor volume, which is difficult to control on the picoliter scale. Images were shading corrected and background subtracted using ImageJ software (v. 1.49S, http://imagej.nih.gov/ij). Downstream analysis of sensor intensities was performed using custom-built software based on LabView (v. 2014, National Instruments, Austin, TX). Sensor intensity data was extracted by generating a matching array of circular regions of interest that were aligned with the locations of the wells in images. A sliding 5-point average was applied to some of the data to reduce noise levels before fitting the data using a linear regression model. The OCR values were calculated as a slope of the fitted regression line divided by the total volume of the sealed well (140 pL). See Supplementary Materials for details of sensor deposition and calibration procedure and data.

### Experimental workflow

The experimental approach encompasses two main steps performed in a technology platform with two modules: Module 1) a microfabricated device for cellular phenotype characterization (Fig. 1A and Supplementary Fig. S1), and Module 2) a cell harvesting module (Fig. 1B). In Module 1, measurements of oxygen consumption rate (OCR) – a proxy of oxidative phosphorylation activity in the cell– are performed on live single cells. Following the OCR measurement, the same individual cells are harvested, lysed and the cellular material is collected in Module 2 for downstream analysis via e.g. RT-qPCR, microarray analysis or next generation sequencing while keeping track of the cell identity and its functional readout. To perform functional assays (oxygen consumption profiling in our study) in Module 1, the individual cells are loaded into optically transparent glass microwells in an array (bottom array) using random seeding (Fig. 1A, and Supplementary Fig. S1) followed by a brief (10–15 minutes) incubation and subsequent washing to remove cells outside the wells. Cells in the microwells (bottom array) are then incubated for 16–24 hours prior to experiments. Following incubation, the individual oxygen consumption rate of each cell is determined by reversibly creating a gas-impermeable chamber of 140 pL via hermetically sealing the bottom array containing single cells with a top array containing a ratiometric optical oxygen sensor covalently attached to the bottom and the walls of the microwells (Fig. 1A and Supplementary Figs 1 and 2)^27^.

After the oxygen consumption experiment is complete the presence of hermetic sealing in the wells was verified by purging the media with 100% nitrogen and observing sensor intensity inside the wells over 30–50 minutes. No change in sensor intensity indicated fully sealed wells. Following the seal test in Module 1, Module 2 is invoked for downstream analysis of the single cells. The top array is removed and the cells are harvested from the bottom array using fluid flow produced via aspiration of the cell culture medium into a microcapillary (Fig. 1B)^44^. To this end a custom-built high-precision diaphragm micro-pump (“pico-pump”, see Supplementary Materials, Supplementary Fig. S3) is used^26^,^27^. The single cell in the microwell is aspirated into the tip of the capillary and transferred into a vial (e.g. PCR tube) (Fig. 1B).

The technology platform enables the incorporation of multiple different optical sensors to measure different analytes simultaneously inside the microwells via mixing (for spectrally different sensors) or spatial separation (for spectrally overlapping sensors) via sensor photopatterning (Supplementary Fig. S4). Moreover, due to the optical compatibility of the bottom array and top array chips (Module 1), the approach is amenable to a broad variety of other live cell imaging assays to correlate other, e.g. intracellular functional, readouts, with the physiologic phenotype and gene expression levels of the same cell.

### Cell harvesting after drawdowns

After completion of drawdowns the lids were removed from the station slowly to avoid cell loss or damage due to fluidic flows. The well substrate was washed gently with warm phosphate buffered saline (PBS) three times to remove debris and detached or dead cells on the outside of the wells. The chip with cells in micro wells was transferred to a harvesting station and placed into a petri dish filled with warm PBS. The harvesting station was built around a modified inverted microscope equipped with a custom-built high-precision computer controlled diaphragm pump (Supplementary Fig. S3) that allows for sub-nanoliter volumes to be aspirated and dispensed in a highly controlled manner^26^. Individual cells were harvested using the pump equipped with a glass micropipette with a 40 μm inner diameter that was optimized to enable aspirating and dispensing individual cells from each well. A low concentration (0.05% v/v) of trypsin solution followed by brief incubation (4–5 minutes) was applied followed by aspirating the cell of interest into the microcapillary connected to the pump. The combination of the trypsin treatment and shear flow was found to result in a high cell harvesting yield (80–95%) without inducing detectable cellular stress response^44^. To harvest a single cell, the cell of interest was brought into the center of the field of view of the microscope objective. Then, the tip of the micropipette was lowered and positioned within 40–70 μm from the cell and the cell was aspirated into the micropipette by the pump. Once the aspiration step was complete, the micropipette tip was raised and aligned with the opening of a PCR tube using a motorized microscope stage, and the cell was dispensed into the tube for lysis. The relative positions of wells containing single live cells on the substrate were recorded during the drawdown and only these wells were subjected to harvesting. The pump and the glass capillary were sterilized with 70% ethanol solution and primed with PBS before harvesting procedure. Each well containing a single cell was inspected visually through a 20x objective lens immediately before harvesting. The cells found in these wells where aspirated one at a time using the pump under visual control and transferred into 50 μL PCR tubes, one cell per tube, in a volume of ~2–4 μL of PBS and snap frozen on dry ice. Some cell types demonstrated rather prominent attachment to the wells. For these cells a pretreatment with 0.25% Trypsin-EDTA solution for 5 minutes followed by a washing step with PBS was used before harvesting to partially detach cells and facilitate harvesting.

### Cell culture preparation

All cells were cultured in T75 tissue culture flasks (Corning, Corning, NY) to approximately 80% confluence under normal cell culture conditions, at which time they were trypsinized, centrifuged at 900 rpm for 3 minutes, and re-suspended in 2 mL of cell growth medium.

The cells were grown at 37 °C, under 5% CO_2_ atmosphere in cell culture flasks using GIBCO^®^ Keratinocyte SFM cell growth medium (Thermo Fisher Scientific, Waltham, MA), supplemented with hEGF (Peprotech, Rocky Hill, NJ) at 2.5 μg/500 mL, BPE (bovine pituitary extract) at 25 mg/500 mL and penicillin/streptomycin solution (Thermo Fisher) at 100 units/100 μg/mL. Prior to loading into wells, cells were detached from the flask bottom using a 0.05% trypsin-EDTA solution and concentrated to 2 · 10^5^ cells/ml in the cell culture medium. For cell loading into the wells 50 μL of the cell suspension were placed onto the well substrates for 15 minutes to allow for cell adhesion to the bottom of substrates. The substrates were then rinsed with 1 mL of PBS, and the petri dish was replenished with 4 mL of the cell culture medium. The cell-seeding substrates with no lid on top were then put in a cell culture incubator for 24 hours prior to the drawdown experiment. For counting purposes, cells were stained with the Hoechst 33343 nuclear DNA stain.

### Hypoxia selection

For hypoxia selection experiments cells were seeded at a density of 1.5 × 10^5^ in T25 flasks. At 48 hours post-seeding, the medium was changed to 5 mL of keratinocyte medium without serum, glucose, l-glutamine, or growth supplements, but containing penicillin and streptomycin. 50 μL of Oxyrase^TM^ (Oxyrase Inc, Mansfield, OH) was added to the medium. The flasks were placed in a CO_2_/nitrogen incubator with O_2_ at 1%, with the culture flask caps loosened to allow for optimal gas exchange. There was no lactate added to the medium. O_2_ concentration in the flask was monitored using a Clark electrode. The electrode was placed in a control flask containing the same medium conditions but without cells. It generally took about 2 hours for the oxygen to get to 0%. At that point, the hypoxia timing was begun. A control flask of cells was counted at this point to determine starting cell numbers.

After hypoxia treatment, the medium was removed from the flasks, the flasks were rinsed twice with sterile 1X PBS and subsequently regular keratinocyte containing 2% serum, growth supplements, l-glutamine and antibiotics. One flask was used for counting total cell numbers. The remaining flasks were incubated for 72 hours post-hypoxia in normal (21% O_2_, 5%CO_2_, 37 °C, 100% relative humidity) culture conditions.

Total number of cells per T25 flask were counted at 24, 48 and 72 hours post-hypoxia. Control flask cells (no hypoxia) were also counted at each time point and passaged if necessary.

## Additional Information

**How to cite this article:** Kelbauskas, L. *et al*. Platform for combined analysis of functional and biomolecular phenotypes of the same cell. *Sci. Rep.* **7**, 44636; doi: 10.1038/srep44636 (2017).

**Publisher's note:** Springer Nature remains neutral with regard to jurisdictional claims in published maps and institutional affiliations.

## Supplementary Material

Supplementary Methods and Materials

## Figures and Tables

**Table 1:** Main functional annotation clusters of the target genes.

Functional group
Positive regulation of chemotaxis
Glycolysis/glucose metabolism
Blood vessel development/morphogenesis
Phosphorylation and phosphorus metabolic process
Cell migration/motility
Phosphorylation regulation
Regulation of apoptosis

The functional groups associated with hypoxia response (blood vessel development and morphogenesis) and energy production (glycolysis/glucose metabolism) were selected as primary targets.

**Table 2:** Total and relative cluster enrichment scores of gene expression by cell type.

Cell type	cCP-A	hCP-A	cCP-B	hCP-B	cCP-C	hCP-C	cCP-D	hCP-D
TES	3.25	5.49	3.16	3.71	4.28	4.94	2.95	2.37
RV	0.59		0.85		0.87		1.25	

A higher Total Enrichment Score (TES) represents lower variability. The relative variability (RV) is a measure of the relative change in variability between the control and hypoxia-resistant strains of the corresponding cell type. The metaplastic cells (CP-A) showed the highest variability level, but also the largest relative decrease in variability after hypoxia-induced selection. The dysplastic cells exhibited lower overall variability and also only slight relative decrease (CP-B and CP-C) or increase (CP-C) in variability.
