# Supplementary material for: Platform for combined analysis of functional and biomolecular phenotypes of the same cell
Source: Sci Rep. 2017 Mar 16;7:44636. doi: 10.1038/srep44636 (PMC5353596; doi:10.1038/srep44636)
Supplement: Supplementary Methods and Materials [file srep44636-s1.pdf]

Platform for combined analysis of functional and biomolecular phenotypes of the same cell

L. Kelbauskas[1], S. Ashili[1], J. Zeng[1], A. Rezaie[1], K. Lee[1], D. Derkach[1], B. Ueberroth[1], W. Gao[1], T. Paulson[2], H. Wang[1], Y. Tian[1], D. Smith[1], B. Reid[2], and Deirdre R. Meldrum[1,*]

[1]Center for Biosignatures Discovery Automation, Biodesign Institute, Arizona State University, Tempe, AZ
[2]Fred Hutchinson Cancer Research Center, Seattle, WA

*Correspondence to: deirdre.meldrum@asu.edu

Supplementary materials

Preparation of the wells and lids arrays

To prepare the sample holder assembly, Falcon® 60 x 15mm not TC-Treated easy-grip style petri dishes (BD Biosciences, San Jose, CA, catalog # 351007,) were placed into a computerized laser cutter (model XL9200, Universal Laser Systems, Scottsdale, AZ) and a single hole with a diameter of 9 mm, centered at the bottom of each petri dish, was cut. After that, the areas around the hole at the bottom of the petri dishes were deburred with a piece of sandpaper, both on the inner and outer side of the dish. The sanding of the petri dish was performed to maximize the contact between the substrate and the petri dish for gluing.

After deburring, the petri dishes were placed in a 1 L beaker containing a 1% micro-90 solution. The beaker was then sonicated for 30 minutes followed by a cascade rinse of the substrates with purified water and spraying with 70% ethanol. After these steps were complete the substrates were air-dried in a laminar flow hood.

Once the petri dishes were dried, a thin line of a general purpose two-component rapid set epoxy gel resin (McMaster-Carr, Los Angeles, CA, catalog # 7670A22,) was deposited around the cut-out hole. A previously cleaned well array substrate was placed face-down (holes facing the inside of the petri dish) and gently pressed to ensure that epoxy completely covered all areas between the well substrate and the petri dish. It was important to ensure that the epoxy was filling all gaps to avoid fluid leaks during experiments. Also, it was crucial to eliminate remaining epoxy on the front (area with wells) and back (area without wells) surface of the

well substrate. This was necessary to prevent the substrates from breaking due to non-uniformly distributed pressure. The well substrates were then placed at 40° C for at least 24 hours to fully cure the glue.

Sensor deposition and calibration

To facilitate covalent bonding of the sensor material to the glass lid surface, lids were cleaned, plasma-treated and functionalized with (3-acryloxypropyl) trimethoxy-silane via vapor deposition. After 18-24 hours of vapor silanization, 1µL of sensor material was applied, via pipette, to the lid array. A custom apparatus was used to apply sufficient pressure to the lid to spread the sensor material uniformly across the array. This apparatus consists of an acrylic base, about 5cm thick, on which sits a circular piece of glass approximately 3cm in diameter. A separate, circular piece of acrylic, also about 5 cm thick, attaches vertically to the base and can be tightened down onto the base via four screws extending through the two acrylic components. Affixed to the underside of the upper acrylic portion of this apparatus is a circular piece of fluorinated glass, about 3cm in diameter. Each lid was placed, with the array facing upward, onto the lower glass piece. The upper fixture was then screwed down onto the base of the apparatus so that the fluorinated glass is applying pressure onto the lid, pressing it into the glass surface of the base. The lid(s) were allowed to thermally cure in a vacuum drying oven (DX400, Yamato, Santa Clara, CA) for approximately 18 hours at 80°C under nitrogen atmosphere (70mmHg/0.01MPa).

While the oxygen sensor phosphorescence signal depends strongly on oxygen concentration ([O_2]) in the surrounding medium, the rhodamine (reference) fluorescence intensity remains constant over a wide range of [O_2] (Supplementary Figure S2A). The sensor response to changes in [O_2] was calibrated using reference solutions containing known concentrations of dissolved oxygen. The reference aqueous solutions were prepared by purging water or cell culture media with N_2+O_2 gas mixtures of the desired oxygen concentrations obtained using a computer-controlled gas manifold (Alicat Scientific, Tucson, AZ). The calibration data were used for oxygen concentration determination in the OCR measurements. Supplementary Figure S2A shows calibration of the sensor in lids submersed in cell media. The measured data points were fit with the Stern-Volmer equation to create a continuous calibration curve (Supplementary Figure S2B):

$$\frac{I_0}{I} = 1 + k_{SV}[C],$$

where I_0 and I are the sensor emission intensities at 0% and unknown concentration of the analyte (oxygen), respectively, k_{SV} is the Stern-Volmer quenching rate coefficient, and [C] is dissolved oxygen concentration.

The goodness of fit supports the assertion that sensor emission intensity is governed by diffusion-limited dynamic quenching by oxygen. With settings for illumination intensity, integration time to achieve a signal-to-noise ratio of at least 4:1, we determined the limits for oxygen concentration detection and quantitation to be 0.1 ppm and 0.2 ppm, correspondingly.

Self-leveling system for hermetic sealing of microwells

Achieving hermetic seals in prior methods of single cell microchip assays[27] has not proved much of an issue, largely due to the small contact surface area over which the relatively large amount of force was distributed. In these original 3x3 arrays, a flat compliant layer proved sufficient in tolerating some small deviations from flatness between substrate and lid, and relatively small amounts of weight (<5 kg) created sufficient force at this lid-substrate interface to seal the 9 wells. However, the much larger surface area of the 15x15 arrays used in this study created problems in sealing with the original methods, requiring novel routes for achieving a truly flat approach of the lid to the substrate, as well as applying greater, more stable mechanical force across the array.

We have developed a pneumatic-based approach involving a pressurized air channel which pressurized the lid during drawdown onto the substrate (Supplementary Figure S7). Experimental setup utilized a vertical piston to move the lid in the Z direction relative to the substrate sitting on the microscope stage. A piston (5 mm OD, 3 mm ID, 50 mm length) was fabricated with a channel running through its center of approximately 3 mm diameter. At the bottom of the piston, a custom-fabricated molar was used as the removable interface between the piston and lid. An aluminum molar tip was placed inside the molar through the top so it sits snugly in the bottom of the molar with the tip portion protruding through the bottom. To effectively pressurize the molar tip, and thereby the lid, the internal molar chamber was sealed using a silicone-based sealant, applied liberally via a pipette tip to the inside of the molar chamber surrounding the molar tip base. A third component of the molar is a double-sided screw with one side screwing into the top of the molar chamber and the other into the piston on

the microscope stage. This screw component was also fabricated with a small vertical channel (250 µm diameter) allowing the pressurized air to flow through the piston and reach the molar tip. Teflon tape was applied to the screw portion going into the molar chamber to keep this screw from loosening. A small O-ring was also screwed on to the component fitting into the piston to prevent leakage from the pressurized channel at the piston-molar interface. The sealant in the molar was allowed to set for 18-24 hours. A calibrated lid was then affixed to the bottom of the molar tip using double-sided tape (3M, St. Paul, MN) and the molar was screwed back into the piston. To achieve hermetic sealing of the array, the airline was turned to a reading of 4.8 atmospheres and the piston/molar lowered to contact the substrate. In this way, slight deviation from a 90° approach of the piston to substrate can be tolerated by the pressure backing the molar tip and, thus, the lid. The combination of this effect stabilizing the lid and the compliant Parylene C layer of the substrate help maximize hermetic sealing across the entire 225 microwell array.

Cell viability

We assessed cell viability after cell loading and incubation using the CalceinAM/Sytox Orange live/dead assay (Thermo Fisher Scientific) (Supplementary Figure S5). We found that >99% of cells loaded in microwells showed enzymatic activity. Five to ten percent of loaded cells divided in the microwells during the incubation time, suggesting excellent cell health and near-normal function after confinement in microwells. We account for these microwells by utilizing transmission brightfield imaging to determine the number of cells in each microwell prior to experiments. Wells containing cells that underwent division during the incubation time were not included in the data analysis.

Gene expression level analysis

<u>RNA isolation and purification from single cells</u>

The single cell RNA was isolated and purified using the ZR-96 Quick-RNA™ kit (ZYMO Research Corp., Irvine, CA, catalog # R1052) following the manufacturer's instructions: 1) 50 µL of RNA Lysis Buffer were

added into the tube containing a single cell and then vortexed briefly. 2) 50 µL 100% ethanol were added to the tube and mixed well. The mixture was transferred to a Silicon-A plate mounted on a collection plate and then centrifuged at 2,500 x g for 5 minutes. The flow-through was discarded. 3) 400 µL of RNA Prep Buffer were added to the plate followed by centrifuging the plate at 2,500 x g for 5 minutes. The flow-through was discarded. 4) 500 µL of RNA Wash Buffer were added to the plate prior to centrifugation of the plate for 5 minutes at 2,500 x g. The flow-through was discarded. 5) Step 4 was repeated. 6) For RNA elution, the Silicon-A Plate was mounted onto an elution plate, and 15 µL of DNase/RNase-free water were added directly to the matrix, then centrifuged for 5 minutes. The eluted RNA can be used immediately or stored at -80° C until further use. The Cover Foil from the kit was used to prevent evaporation.

Reverse Transcription

Reverse transcription (RT) was performed as described elsewhere[44,46]. Briefly, the cDNA synthesis was performed in a total reaction volume of 10 µL using the SuperScript® VILO™ cDNA Synthesis Kit (Thermo Fisher Scientific, catalog # 11754050,) and consisted of the following reagents: 2 µL of 5× VILO Reaction Mix, 1 µL of 10× SuperScript Enzyme Mix, 5 µL of total RNA from a single cell, as well as 2 µL of DEPC-treated water (Ambion, Austin, TX). After gently mixing the tube contents and incubating at 25 °C for 10 min, the cDNA synthesis was performed at 42 °C for 60 min followed by 5 min at 85 °C for inactivation of the reverse-transcriptase.

Pre-amplification

The pre-amplification of cDNA was performed in 3.68 µL of total reaction volume containing the following: 0.666 µL of Single Cell PreAmp Mix (Ambion, catalog # 4458237), 0.77 µL of C1 PreAmp Dilution Reagent (Fluidigm, San Francsico, CA, catalog # 100-5318), 1.56 µL of C1 DNA Dilution Reagent (Fluidigm, catalog # 100-5317), 0.333 of Pooled Primer Mix (500 nM), 0.35 µL of cDNA. The reaction tube was vortexed (MixMate, Eppendorf, Hauppauge, NY) at 2,000 rpm for 30 seconds and then centrifuged for 10 seconds using a bench-top mini centrifuge (MyFuge Mini, Benchmark Scientific, Sayreville, NJ) at the maximum speed. The reaction tube was then placed in a thermal cycler (MJ Mini Personal Thermal Cycler, Bio-Rad, Hercules, CA)

and run under the following cycling program: 95°C for 10 minutes, 25 cycles of: 95°C for 15 seconds and 60°C for 4 minutes. After the reaction was complete, 10 x dilute the final product with DNA Suspension Buffer (Teknova, Hollister, CA, catalog # T0221).

<u>qPCR with BioMarkHD (Fluidigm) platform</u>

1) Assay PreMix: Each Assay PreMix (5 µL) was prepared by adding 2.25 µL of DNA Suspension Buffer (Teknova, catalog # T0221), 2.5 µL of 2X Assay Loading reagent (Fluidigm, catalog # 85000736), 0.25 µL of each PCR primer (100 µM). The Assay PreMix was vortexed vortexed on a MixMate platform at 2,000 rpm for 20 seconds and then centrifuged using a bench-top mini centrifuge (MyFuge Mini, Benchmark Scientific) at the maximum speed for 30 seconds.
2) Sample PreMix was prepared using the following recipe: 3 µL of SsoFast EvaGreen Supermix with Low ROX (Bio-Rad, catalog # 1725211), 0.3 µL of 20X DNA Binding Dye Sample Loading reagent (Fluidigm, catalog # 100-3738), and 2.7 µL of the pre-amplified product. The PreMix was vortexed at 2,000 rpm for 30 seconds and then centrifuged using a bench-top mini centrifuge (MyFuge Mini, Benchmark Scientific) at the maximum speed for 10 seconds.
3) qPCR was performed on the BioMarkHD system (Fluidigm) following the manufacturer’s instructions.

<u>BioMarkHD data pre-processing</u>

The qPCR data generated by the BioMarkHD platform were pre-processed using Fluidigm’s Real-Time PCR Analysis software (version 4.1.3). The limit of detection was set to 24 as a threshold. The automated calling for genes that passed the quality control was adjusted for each gene according to the corresponding melting temperatures. A list of genes that passed QC was exported as an Excel spreadsheet for further analysis as described below.

Statistical and multivariate analyses

The statistical significance for OCR distribution mean difference was determined using the two-tailed non-parametric Kolmogorov-Smirnov test at a significance level of 0.05. The OCR hierarchical cluster analysis was

performed using the group average as a cluster method and correlation distance. A statistical significance test and hierarchical clustering were conducted using OriginPro (version 8.6.0, OriginLab Corp., Northampton, MA) software.

Single cell gene expression level analysis, including PCA, hierarchical clustering and differential gene expression, was performed using the SINGuLAR™ analysis toolset fluidigmSC R library version 3.5.2 (Fluidigm) for R. FluidigmSC was run in R version 3.0.2. Hierarchical clustering (HC) analysis was done using the Pearson correlation method and complete linkage. Principal component analysis (PCA) was performed using the correlation matrix as a distance metric.

The t-SNE analysis was done in R (64 bit, version 3.0.2) using a library developed by J. Donaldson and available in the CRAN repository for download (https://cran.r-project.org/web/packages/tsne/tsne.pdf). The functional gene annotation clustering analysis was performed using the DAVID bioinformatics software package (https://david.ncifcrf.gov/home.jsp)[47,48].

The relative variability within and between the different cell types and strains was quantified based on the difference between the observed probability of cells to occupy a particular cluster vs. the probability to occupy the same cluster by pure chance. The theoretical (random) probability for a cell of type j ($j = 1, .., N$, where $N=4$ *(cell types) x2 (strains)* $= 8$ is the total number of cell types) to occupy cluster i ($i = 1, .., M$, where $M = 10$ is the total number of clusters) of the gene expression data is:

$$p_{j,i} = \frac{N_i}{N_{total}}, \quad \text{(S1)}$$

where N_i is the number of cells in cluster i, and N_{total} is the total number of cells analyzed. Accordingly, the observed probability for a cell of type j to occupy a cluster is:

$$P_{j,i} = \frac{N_{j,i}}{N_{j,total}}, \quad \text{(S2)}$$

where $N_{j,i}$ is the number of type j cells in cluster i, $N_{j,total}$ is the total number of type j cells. The Total Enrichment Score (TES) for cell type j was calculated using the following formula:

$$TES_j = \sqrt{\sum_{i=1}^{M}\left(\frac{p_{j,i}-P_{j,i}}{p_{j,i}}\right)^2} \quad \text{(S3)}$$

The normalized sum of squares of probability differences was used to better account for both the increase and decrease of the observed probabilities with respect to the random chance of cells to occupy clusters. The Relative Variability (RV) was calculated as a ratio of the TES values of the control and hypoxia-resistant strain:

$$RV = \frac{TES_{control}}{TES_{resistant}} \quad \text{(S4)}$$

Experimental setup for Oxygen Consumption Rate (OCR) measurements

The experimental setup used for OCR measurements was described elsewhere[27]. Briefly, the chips were mounted onto a stage of a commercial inverted microscope (Ti, Nikon Instruments, Melville, NY) equipped for epi fluorescence imaging. A cooled EMCCD camera (ProEM, Princeton Instruments, Trenton, NJ) was used as detector. For excitation a multi LED light source (LED4, Thorlabs, Newton, NJ) was utilized. A dichroic mirror (440 dclp, Chroma Corp., Bellows Falls, VT) mounted in the filter turret of the microscope was used to separate emission photons from the excitation light. The emission signal was passed through one of two bandpass filters (BP595/70 and BP650/50, Omega Optical, Brattleboro, VT) for rhodamine and oxygen sensor emissions, respectively. All experiments were performed at 37° C temperature controlled by means of an environmental chamber enclosing the microscope. For sensor imaging a 4X objective lens (PlanApo, NA=0.2, Nikon) was used. The images of the sensor were taken every 30 seconds. The time interval can be adjusted down to sub-seconds to accommodate faster OCR.

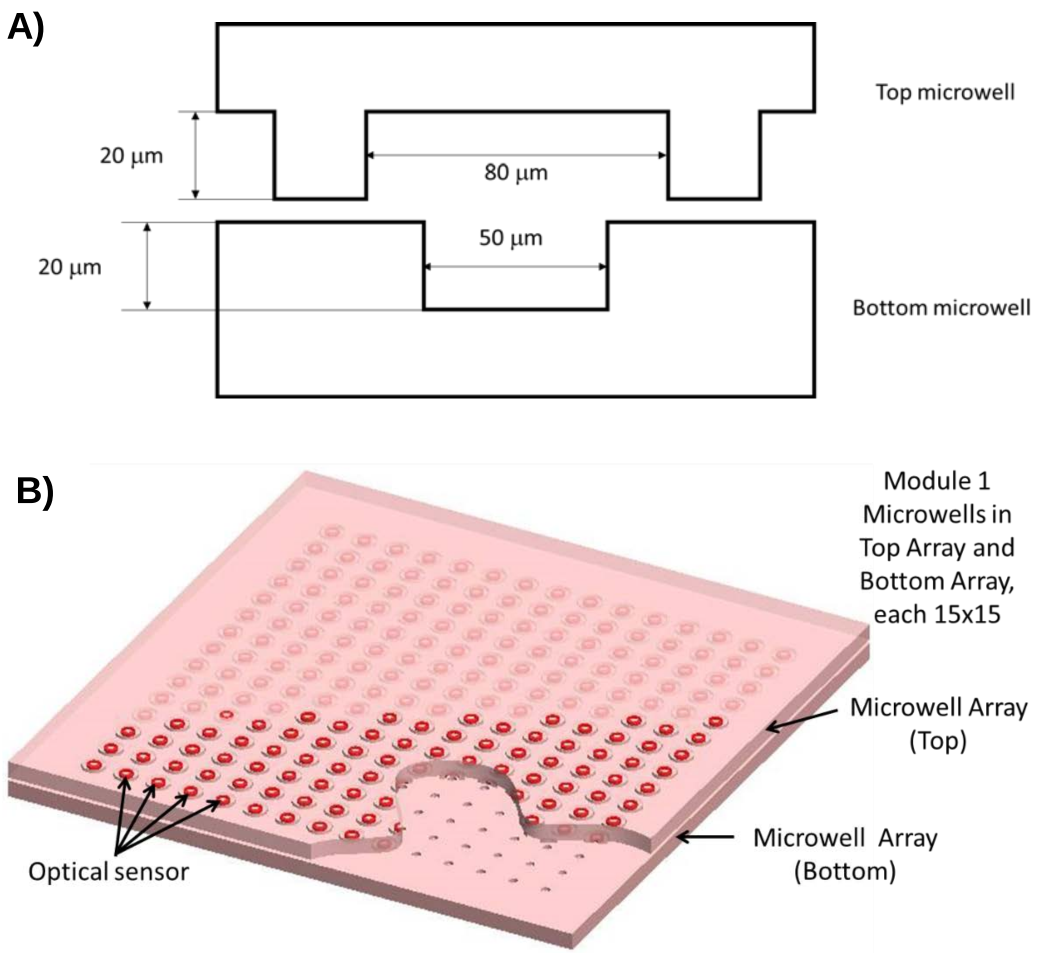

Supplementary Figure S1. Design of the microfluidic device to create hermetically sealed microchambers with integrated optical sensor that hold individual cells during measurements (A). The device consists of a lid (top) and a well (bottom) substrates each containing a matching 15x15 array of lipped (top) or simple wells (bottom) microfabricated using wet-etch photolithographic methods. Red rings represent the optical sensor (B). Panel B is courtesy of Jeff Houkal.

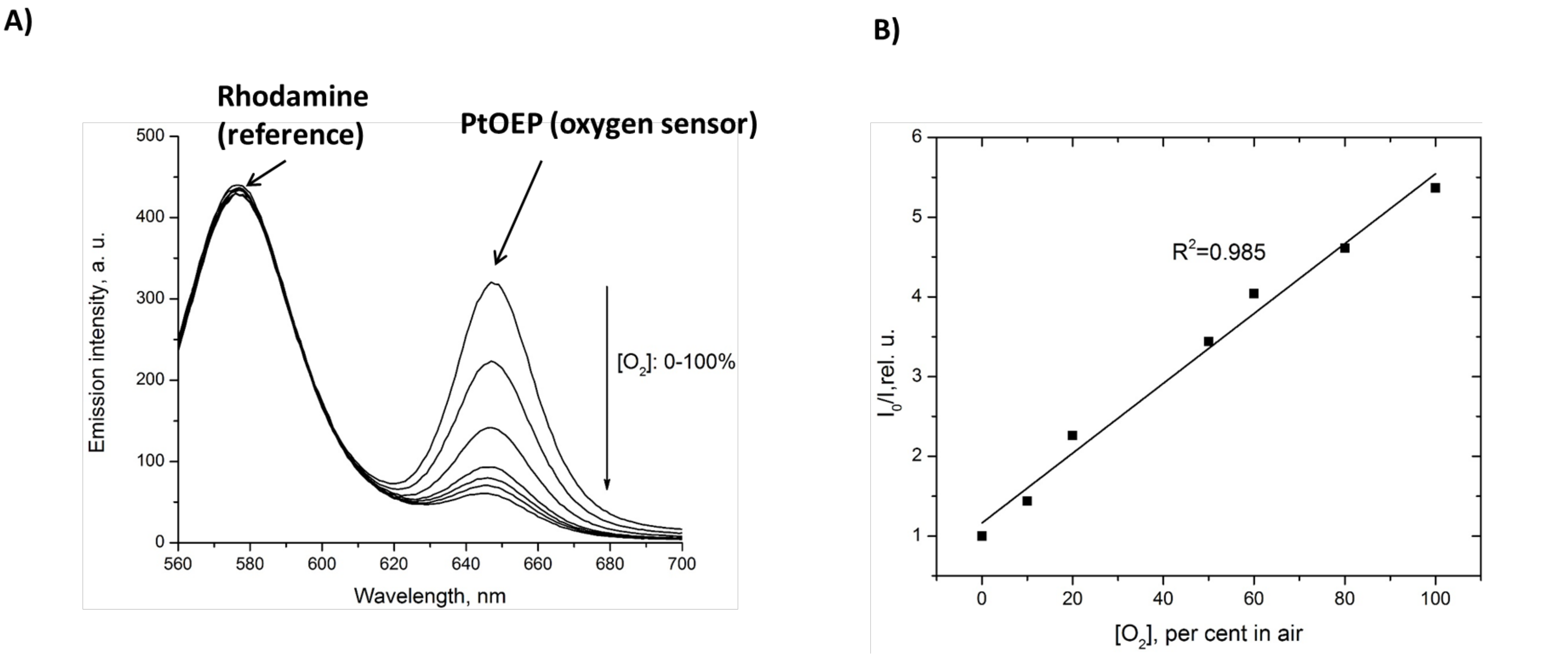

Supplementary Figure S2. Ratiometric oxygen sensor and its response to differing oxygen concentrations A) Sensor emission spectra as a function of oxygen concentration. The emission peak centered at 575 nm is that of the internal reference (Rhodamine 123) which is inert to oxygen. The emission peak at 650 nm represents the sensor signal. The signal decreases as dissolved oxygen concentration increases due to the dynamic quenching of the sensor excited state via a triplet-triplet energy transfer to the molecular oxygen. B) Sensor response calibration fitted with the inverse Stern-Volmer equation. The R^2 value indicates good agreement between the sensor performance and the theoretical values.

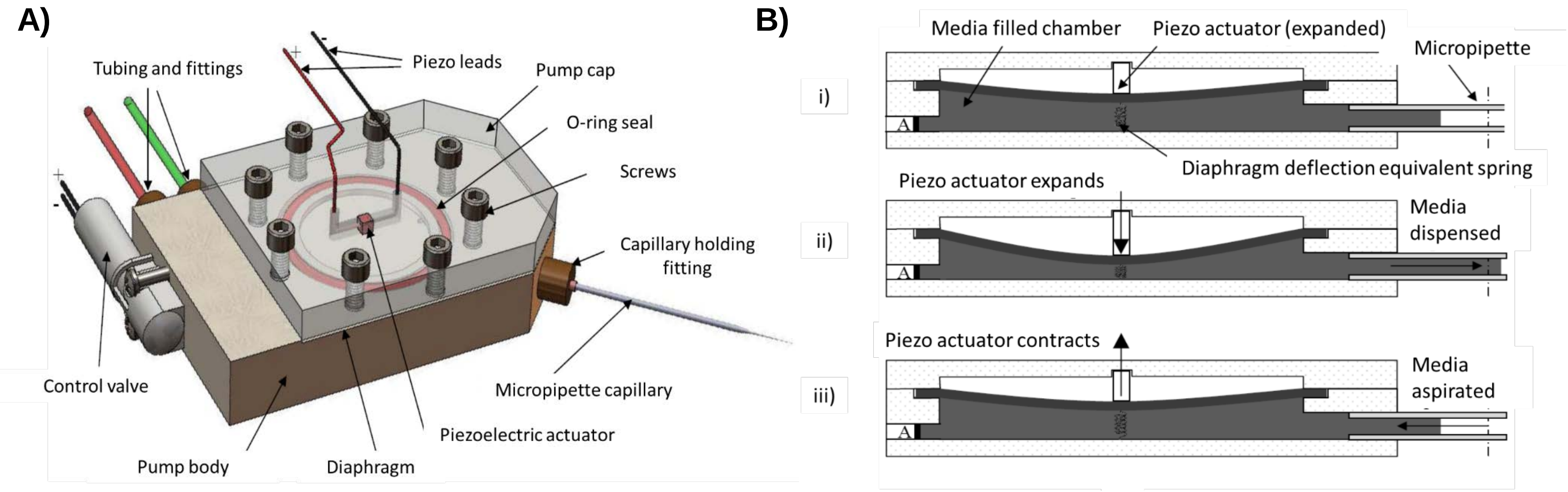

Supplementary Figure S3. Design and principle of operation of the custom high-precision computer controlled pump ("pico-pump) used in the study for retrieving individual cells from wells after the drawdown experiment. A) A 3D rendering of the pico-pump with a tapered microcapillary mounted at the opening (adopted from [20] with permission). B) Working principle of the pico-pump. The pump is connected with another, larger format pump via A for manipulating larger (tens of nL) volumes for priming and bleeding purposes. The pico-pump works by deflecting a thin metal diaphragm (i) via a piezoelectric actuator thus altering the effective volume inside the pump chamber (ii). As a result, sub-nL volumes of liquid can be pushed out (ii) or aspirated (iii) via the micropipette attached to the pump outlet.

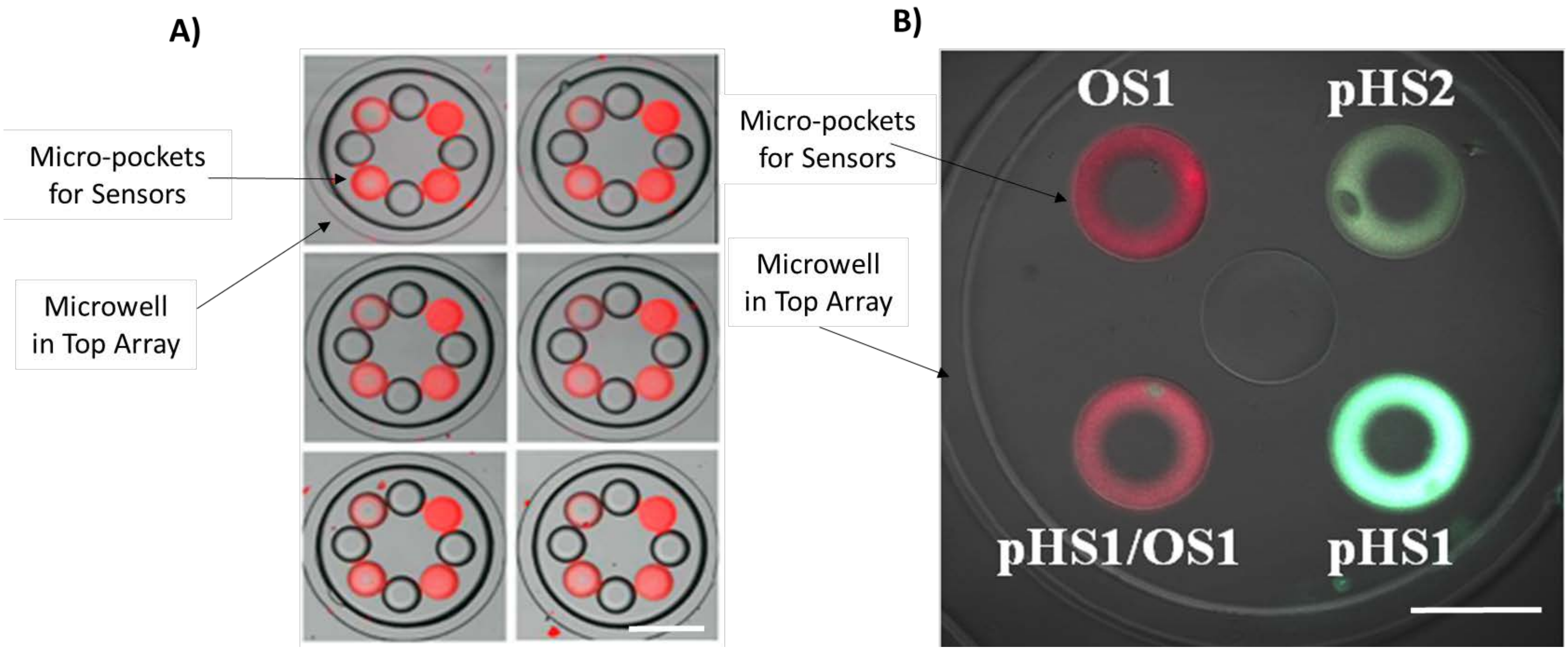

Supplementary Figure S4. Conceptual design of a multi-sensor well for multiparameter functional readouts with spatially separated optical sensors. A) Microwells capable of accommodating up to 8 different optical sensors in spatially separated sub-wells. Scale bar: 200 µm; B) A 5-pocket well containing 4 different sensors for oxygen and pH with different spectral characteristics illustrating the ability of the approach to accommodate multiple sensors per well. OS1-oxygen sensor, pHS2 – pH sensor, pHS1/OS1 – combined oxygen and pH sensor, pHS1 – pH sensor. Scale bar – 50 µm.

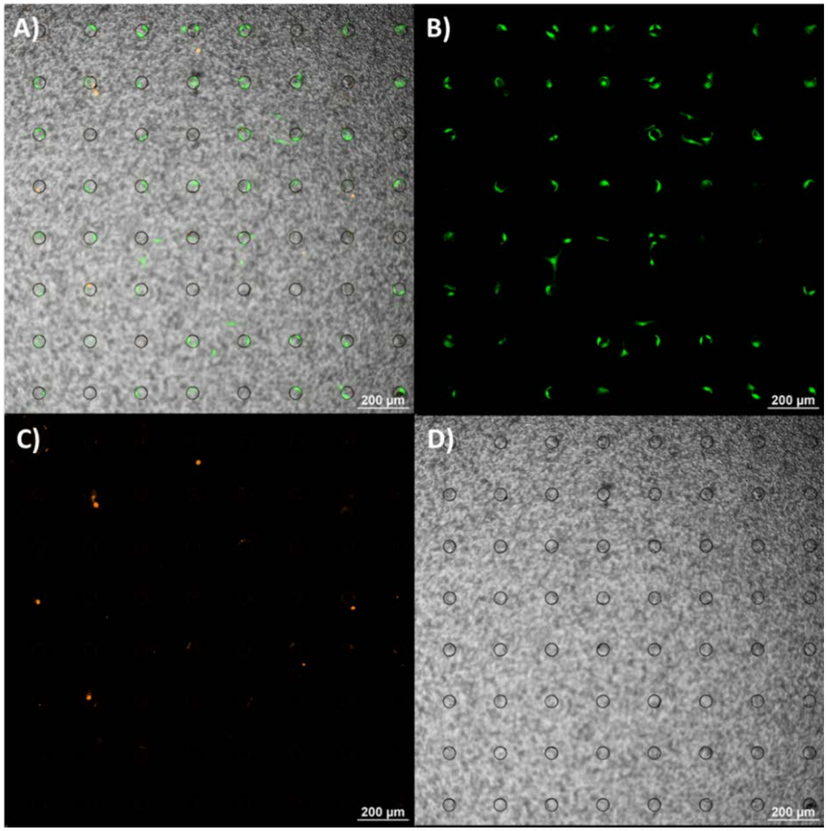

Supplementary Figure S5. Cell viability on well arrays. Prior to imaging cells were seeded (Materials and methods) on the well substrate and incubated for 24 hours under normal culture conditions. The cells were stained using the LIVE/DEAD stain (ThermoFisher Scientific, catalog # L3224,) following the manufacturer's protocol and imaged on an inverted confocal microscope. A) Combined transmission and two-channel (green for live stain and orange for dead stain) confocal fluorescence micrograph. B) Confocal live stain image of the cells in wells. The majority of cells show strong green signal and elongated morphology indicating excellent health. C) Confocal micrograph of the dead stain demonstrated only a few dead cells outside of the wells. D) Transmission micrograph highlighting locations of the wells. Only about 50% (8x8 array) of all wells on a typical substrate are shown.

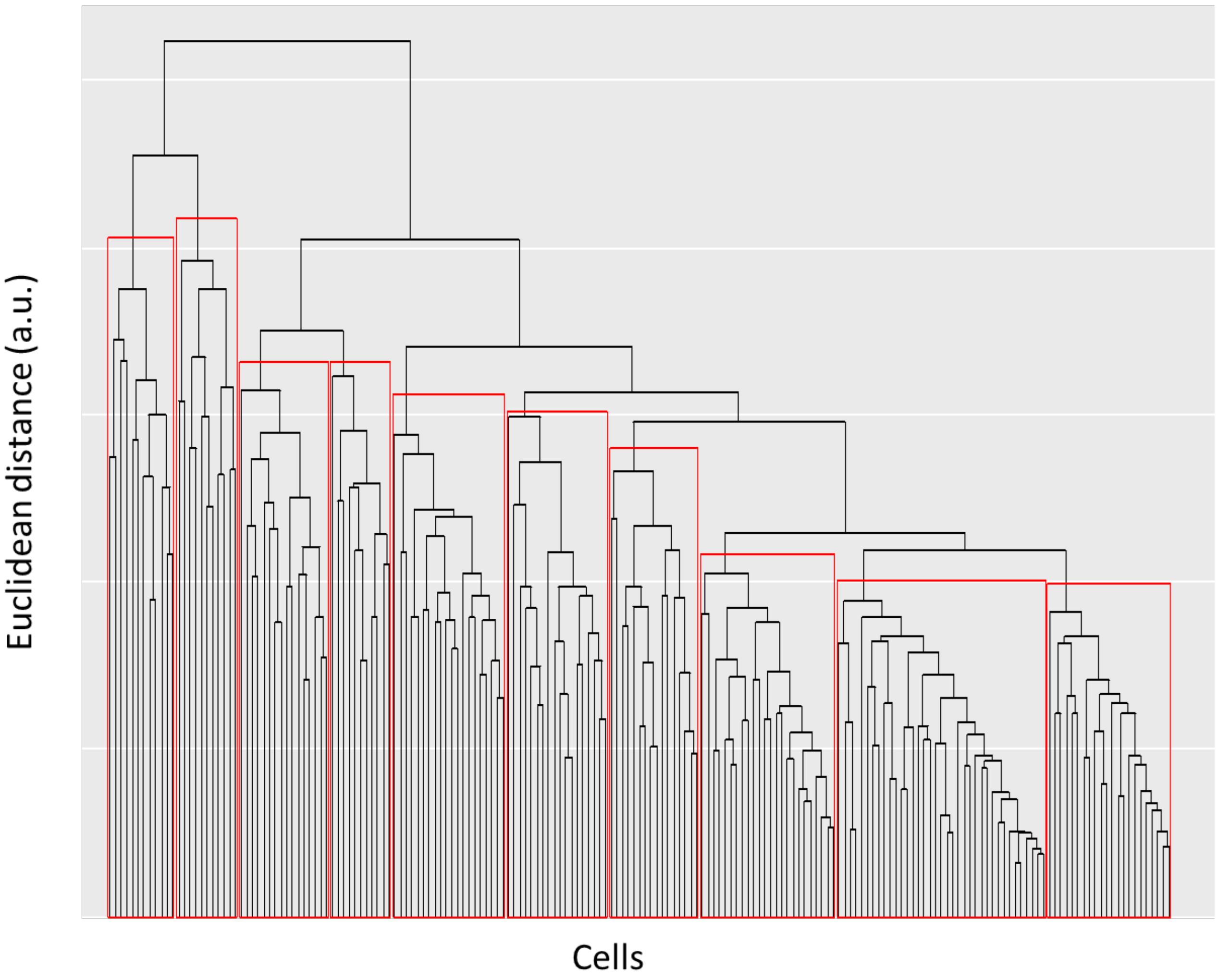

Supplementary Figure S6. Selected sample clusters (red rectangles) from HC data used in determination of variability levels in gene expression profiles from Fig. 4A. The clusters were defined by visual examination of the HC data.

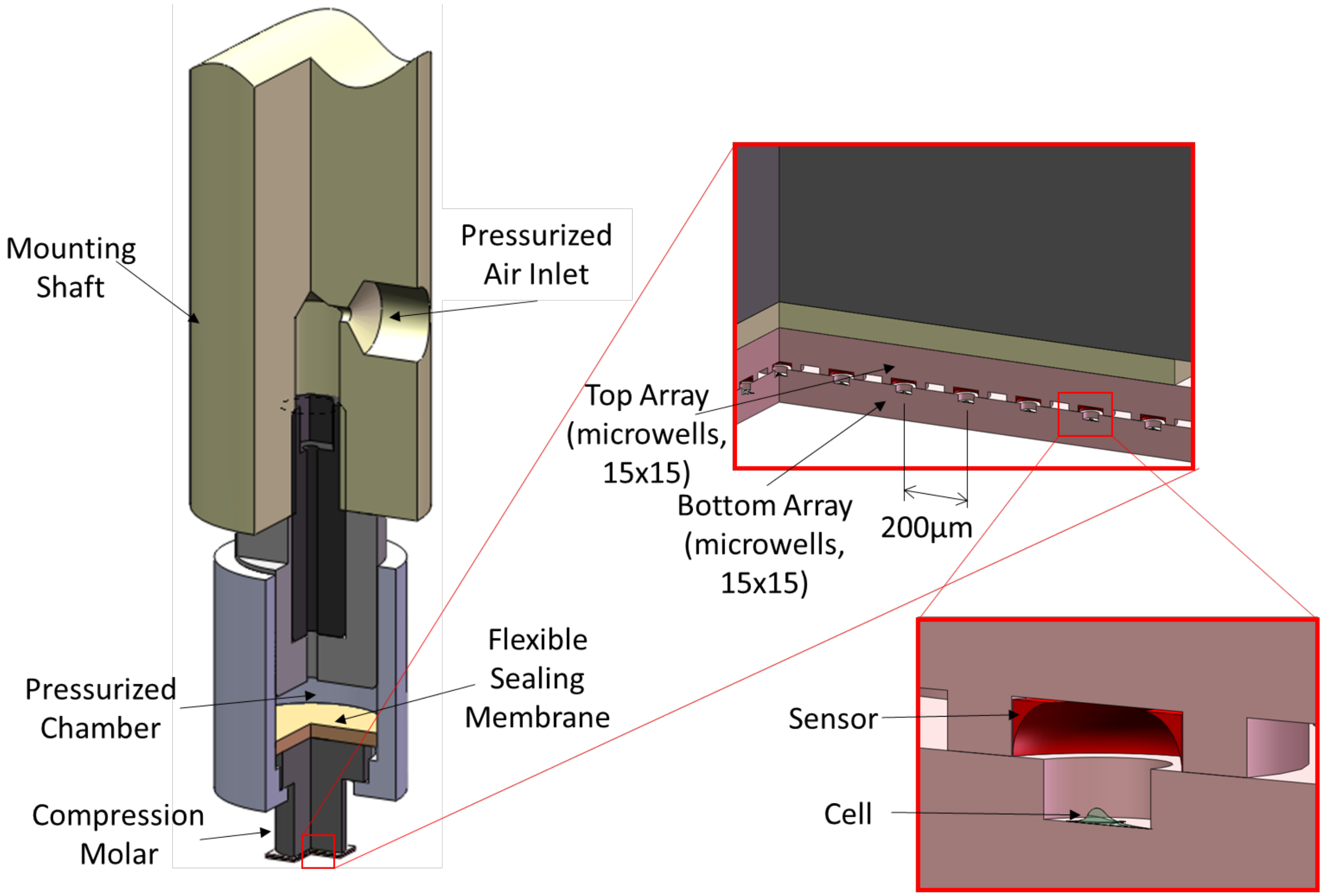

Supplementary Figure S7. Design and working principle of a custom pneumatic molar for producing hermetically sealed microchambers on 15x15 arrays. After the compression molar with a top array attached contacts the bottom array the chamber is pressurized with air. The air pressure is transmitted onto the compression molar and the top and bottom arrays via the flexible sealing membrane creating hermetically sealed volumes containing single cells. The approach provides uniform force distribution across the surface and enables a high level of control of the force that can be dynamically adjusted during the sealing step (schematic courtesy of Jeff Houkal).

Supplementary tables

Supplementary Table S1. Main genotype alterations of the studied cell lines. “wt” – wild type gene, “-“ mutation or loss of heterozygosity

Cell line	Patient Dx	Mutation	DNA content
CP-A	Non-dysplastic metaplasia	p16-/- p53 wt	2.6N
CP-B	High-grade dysplasia	p16-/- p53 -/-	2.2N
CP-C	High-grade dysplasia	p16+/- p53 -/-	2.1N
CP-D	High-grade dysplasia	p16-/- p53 -/-	2.4, 3.6, 4.1N

Supplementary Table S2. Descriptive statistics of the OCR data shown in Figure 3B. SD – standard deviation, CV – coefficient of variation

Cell type	N total	Mean	SD	Sum	CV	Min	Median	Max
cCP-A	24	0.26	0.14	6.21	0.53	0.05	0.23	0.58
hCP-A	24	0.16	0.12	3.93	0.73	0	0.15	0.43
cCP-B	24	0.34	0.38	8.27	1.09	0	0.25	1.64
hCP-B	24	0.16	0.15	3.92	0.94	0	0.13	0.56
cCP-C	22	0.22	0.14	4.74	0.66	0	0.23	0.56
hCP-C	24	0.30	0.57	7.17	1.92	0	0.20	2.95
cCP-D	23	0.39	1.10	8.99	2.81	0	0.11	5.34
hCP-D	23	0.19	0.17	4.29	0.90	0	0.20	0.54

Supplementary Table S3. List of genes used in the study

No.	Gene Symbol	Name
1	CSF3	colony stimulating factor 3 (granulocyte)
2	WWC1	WW and C2 domain containing 1
3	ATP6V1G2	ATPase, H+ transporting, lysosomal 13kDa, V1 subunit G2
4	ADRB2	adrenergic, beta-2-, receptor, surface
5	MTPN	myotrophin; leucine zipper protein 6
6	MAP3K1	mitogen-activated protein kinase kinase kinase 1
7	PDK1	pyruvate dehydrogenase kinase, isozyme 1
8	ALDH2	aldehyde dehydrogenase 2 family (mitochondrial)
9	EDN1	endothelin 1
10	ACSS1	acyl-CoA synthetase short-chain family member 1
11	YY1	YY1 transcription factor
12	LDHA	lactate dehydrogenase A
13	ENO2	enolase 2 (gamma, neuronal)
14	RPSA	ribosomal protein SA pseudogene 9; ribosomal protein SA pseudogene 8; ribosomal protein SA pseudogene 58; ribosomal protein SA pseudogene 19; ribosomal protein SA pseudogene 18; ribosomal protein SA; ribosomal protein SA pseudogene 15; ribosomal protein SA pseudogene 61; ribosomal protein SA pseudogene 29; ribosomal
15	PGK2	phosphoglycerate kinase 2
16	IL6R	interleukin 6 receptor
17	IRF3	interferon regulatory factor 3
18	RLIM	ring finger protein, LIM domain interacting; similar to ring finger protein (C3H2C3 type) 6
19	F2RL1	coagulation factor II (thrombin) receptor-like 1
20	GNA12	guanine nucleotide binding protein (G protein) alpha 12
21	XDH	xanthine dehydrogenase
22	EDN2	endothelin 2
23	MGEA5	meningioma expressed antigen 5 (hyaluronidase)
24	FBP1	fructose-1,6-bisphosphatase 1
25	SNAI1	snail homolog 1 (Drosophila)
26	ALDH3A1	aldehyde dehydrogenase 3 family, memberA1
27	P2RY6	pyrimidinergic receptor P2Y, G-protein coupled, 6
28	HKDC1	hexokinase domain containing 1
29	PDK3	pyruvate dehydrogenase kinase, isozyme 3
30	FOXC2	forkhead box C2 (MFH-1, mesenchyme forkhead 1)
31	TGFBR1	transforming growth factor, beta receptor 1
32	AGER	advanced glycosylation end product-specific receptor
33	KITLG	KIT ligand
34	IRF7	interferon regulatory factor 7
35	SREBF1	sterol regulatory element binding transcription factor 1
36	BMP6	bone morphogenetic protein 6
37	INHBA	inhibin, beta A
38	LEP	leptin
39	LAMA3	laminin, alpha 3
40	VEGFA	vascular endothelial growth factor A
41	ABL1	c-abl oncogene 1, receptor tyrosine kinase
42	PDK2	pyruvate dehydrogenase kinase, isozyme 2
43	FZD8	frizzled homolog 8 (Drosophila)
44	VIPR1	vasoactive intestinal peptide receptor 1
45	PDGFB	platelet-derived growth factor beta polypeptide (simian sarcoma viral (v-sis) oncogene homolog)
46	DOCK2	dedicator of cytokinesis 2
47	CFTR	cystic fibrosis transmembrane conductance regulator (ATP-binding cassette sub-family C, member 7)
48	IL8	interleukin 8
49	HNRNPAB	heterogeneous nuclear ribonucleoprotein A/B
50	CXCL1	chemokine (C-X-C motif) ligand 1 (melanoma growth stimulating activity, alpha)
51	ENG	endoglin
52	IL18	interleukin 18 (interferon-gamma-inducing factor)
53	MKL1	megakaryoblastic leukemia (translocation) 1
54	HIF1A	hypoxia inducible factor 1, alpha subunit (basic helix-loop-helix transcription factor)
55	COX6B2	cytochrome c oxidase subunit VIb polypeptide 2 (testis)
56	NDUFS4	NADH dehydrogenase (ubiquinone) Fe-S protein 4, 18kDa (NADH-coenzyme Q reductase)
57	FGFR1	fibroblast growth factor receptor 1
58	PIK3CD	phosphoinositide-3-kinase, catalytic, delta polypeptide
59	SDPR	serum deprivation response (phosphatidylserine binding protein)
60	XYLT1	xylosyltransferase I
61	MX1	myxovirus (influenza virus) resistance 1, interferon-inducible protein p78 (mouse)
62	ADAM12	ADAM metallopeptidase domain 12
63	COL4A1	collagen, type IV, alpha 1
64	KLRC1	killer cell lectin-like receptor subfamily C, member 1
65	LDHB	lactate dehydrogenase B
66	PRKCE	protein kinase C, epsilon
67	SERPINE1	serpin peptidase inhibitor, clade E (nexin, plasminogen activator inhibitor type 1), member 1
68	SMAD7	SMAD family member 7
69	EGR1	early growth response 1
70	SMAD2	SMAD family member 2
71	MKL2	MKL/myocardin-like 2
72	KLF4	Kruppel-like factor 4 (gut)
73	TNFSF11	tumor necrosis factor (ligand) superfamily, member 11
74	KLF2	Kruppel-like factor 2 (lung)
75	IGF2BP1	insulin-like growth factor 2 mRNA binding protein 1
76	TP53	tumor protein p53
77	EGR2	early growth response 2
78	LDHC	lactate dehydrogenase C
79	TXN	thioredoxin
80	SFTPA1	surfactant protein A1B; surfactant protein A1
81	STAT6	signal transducer and activator of transcription 6, interleukin-4 induced
82	S1PR2	sphingosine-1-phosphate receptor 2
83	PDK4	pyruvate dehydrogenase kinase, isozyme 4
84	IFNAR1	interferon (alpha, beta and omega) receptor 1
85	GAPDH	glyceraldehyde-3-phosphate dehydrogenase-like 6; hypothetical protein LOC100133042; glyceraldehyde-3-phosphate dehydrogenase
86	CAT	catalase
87	GNAO1	guanine nucleotide binding protein (G protein), alpha activating activity polypeptide O
88	ETV4	ets variant 4
89	PLAUR	plasminogen activator, urokinase receptor
90	CTGF	connective tissue growth factor
91	MAPK1	mitogen-activated protein kinase 1
92	SLC2A1	solute carrier family 2 (facilitated glucose transporter), member 1
93	CSF2	colony stimulating factor 2 (granulocyte-macrophage)
94	SRF	serum response factor (c-fos serum response element-binding transcription factor)
95	PKM2	Pyruvate Kinase, Muscle
96	ACKR2	Atypical Chemokine Receptor 2

Supplementary Table S4. Functional annotations of genes used in the study. FE – fold enrichment, Benj. – Benjamini-Hochberg correction of p values for multiple comparisons, FDR – false discovery rate. Only annotation clusters with corrected p values of >=0.05 are shown.

Category	Term	Count	%	PValue	Genes	FE	Benj.	FDR
GOTERM_BP_FAT	GO:0050921~positive regulation of chemotaxis	7	7.45	2.88E-08	IL8, PDGFB, EDN2, VEGFA, F2RL1, IL6R, AGER	36.28	2.64E-06	4.83E-05
GOTERM_BP_FAT	GO:0048514~blood vessel morphogenesis	12	12.77	1.29E-07	HIF1A, IL8, CTGF, SMAD7, IL18, TGFBR1, VEGFA, EDN1, FOXC2, MKL2, SRF, ENG	8.55	8.89E-06	2.17E-04
GOTERM_BP_FAT	GO:0042325~regulation of phosphorylation	15	15.96	1.95E-06	CSF2, PDGFB, SMAD7, EDN2, TGFBR1, EDN1, KITLG, IL6R, PRKCE, S1PR2, INHBA, ADRB2, NDUFS4, MAP3K1, ENG	4.84	8.97E-05	3.28E-03
GOTERM_BP_FAT	GO:0006096~glycolysis	7	7.45	5.91E-07	LDHC, LDHB, LDHA, HKDC1, ENO2, GAPDH, PGK2	22.39	3.06E-05	9.94E-04
GOTERM_BP_FAT	GO:0006007~glucose catabolic process	7	7.45	2.10E-06	LDHC, LDHB, LDHA, HKDC1, ENO2, GAPDH, PGK2	18.14	9.15E-05	3.54E-03
GOTERM_BP_FAT	GO:0019320~hexose catabolic process	7	7.45	5.88E-06	LDHC, LDHB, LDHA, HKDC1, ENO2, GAPDH, PGK2	15.25	1.87E-04	9.88E-03
GOTERM_BP_FAT	GO:0046365~monosaccharide catabolic process	7	7.45	6.95E-06	LDHC, LDHB, LDHA, HKDC1, ENO2, GAPDH, PGK2	14.82	2.17E-04	1.17E-02
GOTERM_BP_FAT	GO:0046164~alcohol catabolic process	7	7.45	1.49E-05	LDHC, LDHB, LDHA, HKDC1, ENO2, GAPDH, PGK2	12.99	4.12E-04	2.51E-02
GOTERM_BP_FAT	GO:0044275~cellular carbohydrate catabolic process	7	7.45	1.97E-05	LDHC, LDHB, LDHA, HKDC1, ENO2, GAPDH, PGK2	12.38	5.26E-04	3.31E-02
UP_SEQ_FEATURE	domain:Histidine kinase	4	4.26	1.11E-06	PDK1, PDK2, PDK3, PDK4	162.66	2.15E-04	1.54E-03
GOTERM_BP_FAT	GO:0016477~cell migration	11	11.70	1.31E-05	DOCK2, HIF1A, IL8, PDGFB, CTGF, EDN2, TGFBR1, KITLG, IL6R, SRF, ENG	5.99	3.72E-04	2.20E-02
GOTERM_BP_FAT	GO:0048762~mesenchymal cell differentiation	5	5.32	3.45E-04	HIF1A, EDN1, FOXC2, KITLG, HNRNPAB	14.74	5.70E-03	5.79E-01
GOTERM_BP_FAT	GO:0042981~regulation of apoptosis	15	15.96	7.27E-04	CSF2, TGFBR1, TP53, IL6R, PRKCE, MAPK1, INHBA, ADRB2, MAP3K1, VEGFA, FOXC2, CAT, ABL1, MKL1, MX1	2.80	1.04E-02	1.22E+00
GOTERM_BP_FAT	GO:0016310~phosphorylation	16	17.02	2.08E-04	PDK1, PDK2, FGFR1, SMAD7, PDK3, TGFBR1, PDK4, PIK3CD, SMAD2, PRKCE, S1PR2, MAPK1, NDUFS4, MAP3K1, ABL1, PGK2	3.01	3.66E-03	3.50E-01
UP_SEQ_FEATURE	binding site:NAD or substrate	3	3.19	2.32E-04	LDHC, LDHB, LDHA	122.00	1.78E-02	3.22E-01
GOTERM_BP_FAT	GO:0045860~positive regulation of protein kinase activity	8	8.51	6.60E-04	S1PR2, ADRB2, PDGFB, MAP3K1, EDN2, TGFBR1, EDN1, KITLG	5.39	9.88E-03	1.10E+00
GOTERM_BP_FAT	GO:0001569~patterning of blood vessels	4	4.26	3.36E-04	VEGFA, EDN1, FOXC2, ENG	28.63	5.60E-03	5.63E-01
GOTERM_BP_FAT	GO:0001934~positive regulation of protein amino acid phosphorylation	5	5.32	0.002792	CSF2, TGFBR1, KITLG, IL6R, ENG	8.44	2.98E-02	4.59E+00
GOTERM_BP_FAT	GO:0030324~lung development	5	5.32	0.004097	CTGF, TGFBR1, VEGFA, SMAD2, CFTR	7.59	4.10E-02	6.67E+00
GOTERM_BP_FAT	GO:0043065~positive regulation of apoptosis	9	9.57	0.007298	INHBA, MAPK1, ADRB2, MAP3K1, TGFBR1, TP53, MX1, ABL1, PRKCE	3.15	6.44E-02	1.16E+01
GOTERM_BP_FAT	GO:0042089~cytokine biosynthetic process	3	3.19	0.004837	IL18, IRF3, IFNAR1	28.18	4.69E-02	7.83E+00

Table S5. Cluster enrichment score calculation. p - probability for a cell to fall into a cluster by pure chance, #CP-* - number of cells of type CP-*, where * denotes A through D, in a cluster, P – observed probability of a cell to fall into a cluster.

Cluster ID	Cluster size	p	#cCP-A	P	#cCP-B	P	#cCP-C	P	#cCP-D	P	#hCP-A	P	#hCP-B	P	#hCP-C	P	#hCP-D	P
1	22	0.118	0	0.000	5	0.208	0	0.000	6	0.273	0	0.000	4	0.167	0	0.000	7	0.304
2	37	0.198	15	0.625	4	0.167	0	0.000	0	0.000	10	0.417	3	0.125	1	0.042	4	0.174
3	24	0.128	0	0.000	0	0.000	12	0.545	0	0.000	1	0.042	0	0.000	11	0.458	0	0.000
4	16	0.086	2	0.083	3	0.125	0	0.000	5	0.227	2	0.083	2	0.083	0	0.000	2	0.087
5	18	0.096	4	0.167	0	0.000	6	0.273	0	0.000	2	0.083	0	0.000	5	0.208	1	0.043
6	20	0.107	0	0.000	6	0.250	0	0.000	5	0.227	0	0.000	4	0.167	0	0.000	5	0.217
7	16	0.086	1	0.042	2	0.083	0	0.000	3	0.136	0	0.000	7	0.292	1	0.042	2	0.087
8	11	0.059	0	0.000	0	0.000	3	0.136	1	0.045	0	0.000	0	0.000	6	0.250	1	0.043
9	12	0.064	2	0.083	0	0.000	1	0.045	0	0.000	9	0.375	0	0.000	0	0.000	0	0.000
10	11	0.059	0	0.000	4	0.167	0	0.000	2	0.091	0	0.000	4	0.167	0	0.000	1	0.043
